# Commentary: Neonatal testicular torsion: refining the role of Doppler and contralateral orchidopexy

**DOI:** 10.3389/fruro.2026.2004112

**Published:** 2026-10-27

**Authors:** Cyrine Saadi, Souha Laarif, Aida Daib, Youssef Hellal

**Affiliations:** Department of Pediatric Surgery, Habib Thameur Hospital, Tunis, Tunisia

**Keywords:** contralateral testis, doppler ultrasonography, neonatal testicular torsion, orchidopexy, perinatal torsion

We read with great interest the recent study by Sun et al. (4) reporting a 14-year single-center experience in neonatal testicular torsion (NTT). This series provides valuable data on a rare condition in which delayed recognition frequently results in testicular loss. We would like to highlight two aspects that may help contextualize the interpretation of their findings.

First, the absence of Doppler flow in 28 of 29 unilaterally affected testes (96.55%) should be interpreted cautiously. Because this was a surgically confirmed cohort, predominantly comprising non-viable testes, these data demonstrate the association between absent flow and established ischemia but cannot establish the diagnostic sensitivity of Doppler ultrasonography for early NTT. Importantly, preserved Doppler flow does not necessarily exclude torsion in the neonatal period. Cases of asynchronous perinatal torsion have been reported, in which apparently preserved preoperative Doppler flow preceded the diagnosis of torsion at exploration (1). Therefore, Doppler ultrasonography should remain an adjunct to clinical assessment rather than a prerequisite for exploration when clinical suspicion is high.

Second, the absence of contralateral torsion during follow-up should not be interpreted as evidence of the preventive efficacy of contralateral orchidopexy. In the present series, 23 of 29 patients with unilateral torsion underwent contralateral fixation, with no subsequent contralateral torsion. However, the absence of a comparable unfixed group prevents an assessment of whether fixation reduced this risk. Contemporary practice remains heterogeneous; a recent national analysis of pediatric testicular torsion demonstrated substantial variation in contralateral fixation and highlighted the limited evidence supporting this practice (2).

The timing of torsion further complicates interpretation. Age at diagnosis does not necessarily indicate when torsion occurred, although this distinction may be crucial for understanding the possibility of salvage. A large literature review including 1,336 patients demonstrated the very high rate of testicular loss associated with NTT while also identifying asynchronous torsion as an important concern for the contralateral testis (3). In the series of Sun et al., the few viable testes included low-degree torsions with restoration of perfusion after detorsion. These observations raise the possibility that salvage depends not simply on the interval between diagnosis and surgery but also on recognizing a recent or atypical torsion before irreversible ischemia develops.

These considerations do not diminish the contribution of Sun et al. Rather, they emphasize that NTT management extends beyond testicular salvage. Prompt clinical recognition, avoidance of unnecessary diagnostic delay, appropriate use of Doppler, and careful consideration of contralateral protection remain central. Given the rarity of NTT, multicenter registries incorporating presumed timing of torsion, standardized Doppler findings, degree of torsion, treatment delay, and long-term testicular outcomes could help refine current management strategies.
